# Assessment of Membrane Fluidity Fluctuations during Cellular Development Reveals Time and Cell Type Specificity

**DOI:** 10.1371/journal.pone.0158313

**Published:** 2016-06-30

**Authors:** Pakiza Noutsi, Enrico Gratton, Sahraoui Chaieb

**Affiliations:** 1 Division of Biological and Environmental Sciences and Engineering, King Abdullah University of Science and Engineering, Thuwal, KSA; 2 Laboratory of Fluorescence Dynamics, Biomedical Engineering Department, University of California Irvine, Irvine, California, United States of America; 3 Lawrence Berkeley National Laboratory, 1 cyclotron road, Mailstop 6R-2100, Berkeley, CA 94720, United States of America; Medical College of Wisconsin, UNITED STATES

## Abstract

Cell membrane is made up of a complex structure of lipids and proteins that diffuse laterally giving rise to what we call membrane fluidity. During cellular development, such as differentiation cell membranes undergo dramatic fluidity changes induced by proteins such as ARC and Cofilin among others. In this study we used the generalized polarization (GP) property of fluorescent probe Laurdan using two-photon microscopy to determine membrane fluidity as a function of time and for various cell lines. A low GP value corresponds to a higher fluidity and a higher GP value is associated with a more rigid membrane. Four different cell lines were monitored such as hN2, NIH3T3, HEK293 and L6 cells. Membrane fluidity was measured at 12h, 72h and 92 h. Our results show significant changes in membrane fluidity among all cell types at different time points. GP values tend to increase significantly within 92 h in hN2 cells and 72 h in NIH3T3 cells and only at 92 h in HEK293 cells. L6 showed a marked decrease in membrane fluidity at 72 h and starts to increase at 92 h. As expected, NIH3T3 cells have more rigid membrane at earlier time points. On the other hand, neurons tend to have the highest membrane fluidity at early time points emphasizing its correlation with plasticity and the need for this malleability during differentiation. This study sheds light on the involvement of membrane fluidity during neuronal differentiation and development of other cell lines.

## Introduction

Membrane fluidity is one of the major macroscopic biophysical properties characterizing cellular membranes. Several studies pointed at its implication during cellular events such as endocytosis, membrane fusion and importantly during development [1–11]. Yet none of them was done in a systematic manner. Differentiation of stem cells in vitro resembles to a certain extent embryo development. Interestingly, lipids such as ceramide are involved in stem cell differentiation [12]. During this process prominent morphological changes occur. The capacity of cells such as neurons to change their structure requires high plasticity and is thought to be driven by a set of cytoskeletal proteins [13–17].

In this work we aim to determine membrane fluidity changes during cell development as well as during neuronal precursor development. We also studied 3 other cell lines that are different in their differentiation potential. Cells monitored are L6, NIH3T3 and HEK293. The question addressed here is whether fluidity changes is time dependent, unique to certain cells such as neurons and whether it can be correlated with the type and function of cells and their capacity to undergo drastic morphological changes especially at early time points (h). Fibroblasts, myoblasts and HEK293 cells are good candidates as they serve different functions. Fibroblasts produce cellular matrix and have a structural role. Myoblasts constitute muscular tissue and HEK293 are human embryonic kidney cells. Accordingly, we aim to do comparative analysis with hN2 cells in a time dependent manner. hN2 cells showed most prominent morphological changes 72 hours post seeding. Structural changes include formation of extensive neurites and clustering of cells resembling neurons. Based on the above observations, we chose two other time points to measure GP; 12h and 92h respectively. Cells at the indicated time points showed either different or similar morphological structure compared to the ones at 72h. We aimed to study membrane fluidity before cells change their shape, at the most drastic morphological changes and during time points at which cells didn’t exhibit any further structural differentiation. L6, HEK293 and NIH3T3 cells obtain stable morphology characteristic to their type within 72h and showed different or stable structure at 12h and 92 h respectively. Accordingly, we monitored their membrane fluidity at the indicated time points.

Our results rely on membrane fluidity as an index or marker that reflects cellular plasticity potential. In this regard we are the first to use fluorescence generalized polarization scanning approach via 2-photon microscope to assess membrane fluidity in live cells in a systematic manner. This technique has been widely used to determine dynamic properties of membrane lipids [18–20]. This information can be obtained from the spectroscopic properties of some water sensitive fluorescent probes. One example of such probes is 2-dimethylamino-6-laurylnaphalene (Laurdan), a lipophilic probe, which is sensitive to membrane polarity and is widely used for membrane fluidity studies. Generalized Polarization (GP) is a quantitative spectroscopic measure of membrane fluidity changes at microscopic levels based on spectral changes rather than on polarization changes. Therefore, GP measurements do not require polarizers to obtain information on membrane fluidity. Also turbidity of live cells does not hinder GP calculations. Light scattering is not significant in this case and polarization corrections are not required. On the other hand, the advantages of using 2-photon excitation include less photo-bleaching, accurate depth’s resolution and the possibility to excite Laurdan without using UV lasers. The spectroscopic properties of Laurdan are affected by the composition and dynamics of its local surroundings. Its spectral shift in its emission spectrum is caused by a change in the membrane’s fluidity. A blue emission indicates a more rigid phase whereas a green emission indicates a more fluid phase as determined by its GP value which is sensitive to the lipid packing [18]. Laurdan is especially useful for detecting lipid phase state coexistence since its spectrum is dependent on water penetration into the membrane bilayer and thus indirectly determine membrane’s fluidity [21–23].

Cell membranes mark the boundaries of live cells and their organelles and play an important role in cellular communications and functions [24, 25]. Cellular membranes are dynamic structures in which lipids are constantly being added or removed and vary with time according to the membrane’s type [26]. Accordingly, membrane fluidity changes, during cellular development, can be a good indication of the mechanical behavior of the cell membrane.

Cells harbor different mechanical properties at their membrane and are specific to their types or population. Several researchers pointed at the mechanical signature of cells in order to isolate valuable cells from mixed populations, e.g., circulating stem cells and tumor cells [27–30]. Furthermore, biophysical properties such as membrane’s capacitance and membrane’s play a key role in determining the fate of stem cell differentiation to neurons or other cell types, e.g., bone or fat [31] and will continue to be used in diagnostic assays as well as during cell injections for targeted delivery. Therefore, fluidity assessment in live cells can also be implemented as a biophysical marker.

Here, we studied membrane fluidity changes in four cell lines. Our results on hN2 and HEK293 cells at different time points, showed a significant decrease in fluidity with lower values at 92 h. Similar results were obtained in NIH3T3 cells, however lower fluidity values were observed at 72 h. In L6 cells membrane fluidity increased with time to reach maximum values at 72 h. At 92 h the cell’s membranes became more rigid. This might indicate a stabilization of the cellular phenotype at that time point. Furthermore, among the four cell lines studied so far, hN2 cells have the highest membrane fluidity at early time points. Our data give an indication of the role of membrane fluidity in cellular development.

Our results also show that changes in cellular morphology is accompanied with GP changes and highlight the role of membrane’s fluidity during structural plasticity or structural modification. hN2 cells undergo the most drastic morphological changes reflected by the appearance of prominent neurites. The other cells types did not show drastic structural modifications probably because they serve different functions. Their membrane is more rigid than that of hN2 cells at early time points however. Our measurements indicate clearly the early involvement of membrane fluidity in neuronal development and structural malleability. The higher the demand for structural modification the larger is the membrane’s fluidity. Therefore, our study emphasizes the correlation of membrane’s fluidity with cellular function and cells’ type.

## Materials and Methods

### Cell culture and treatments

hN2 cells were grown at 37 C in 5% CO_2_ in AB2 basal neural medium supplemented with 2% ANS neural medium, 1% L- glutamine and penstreptomycin and 10μg/μl Leukemia inhibitory factor (LIF). Cells were plated on a 35 mm Fischer Scientific glass-bottom petri dishes coated with Matrigel. NIH3T3, HEK293 and L6 cells were grown at 37C in 5% CO2 in Dulbecco’s modified Eagle’s medium (Invitrogen, Carlsbad, CA) supplemented with 10% fetal bovine serum, 1% Pen-Strep, and 2.5 mL of 1 M HEPES. Freshly split cells were plated onto 35-mm Fischer scientific glass-bottom dishes coated with Matrigel. The membrane dye Laurdan (6-dodecanoyl-2-dimethylamino naphthalene; Invitrogen) was dissolved in dimethylsulfoxide (DMSO), and 2.5 mM stock solution was prepared and added to the cell dishes at final concentration of 10μM. Cells were grown devoid of Laurdan and incubated with the dye prior to imaging. Since growth media can have an effect on GP measurements and produce artifacts, due to the presence of serum that contains lipids which can interfere with the probe’s fluorescence, we used serum free media. To avoid autofluorescence and further ensuing corrections, we used a high enough Laurdan concentration of 10μM [32].

### Two photon scanning fluorescence microscope for Laurdan GP measurements

Two-photon excitation fluorescence measurements were performed using an Olympus FV1000 inverted microscope (Olympus, USA) coupled with a MaiTai HP Ti:Sapphire (Newport, USA) laser. A DeepSea unit is used to compensate the group velocity dispersion and an acoustic optical modulator that controls the laser power. The excitation wavelength is 780 nm for Laurdan. This NIR laser beam is expanded in order to fulfill the back aperture of the objective lens (60x water, NA = 1.2, Olympus, USA). Images were acquired by the same objective with Olympus Photon-counting mode. Two bandpass filters at (410–470 nm) and (470–530 nm) before PMTs were used to detect the 2 emission bands of Laurdan. In each pixel up to 4000 photons are collected.

Cells were kept in Temperature and CO_2_ monitored chamber controllers to maintain optimum physiological conditions and to avoid any biased changes in membrane fluidity. Membrane fluidity changes were determined using Generalized Polarization spectroscopic property of Laurdan. This method relies on the use of GP function and two-photon excitation. Parasassi et al. (1990) introduced the concept of GP in the studies of lipid order of model systems [33], which was further extended on cell membranes using the 2-photon scanning microscopy approach [32]. The GP imaging method that we propose is the extension to microscopy of a well-established technique to study membrane structures and dynamics. Laurdan excitation and emission spectra are extremely sensitive to the polarity and to the dipolar dynamics of the environment. Laurdan can be used to determine if a membrane is in a gel or a liquid-crystalline phase state [34]. It shows a 50 nm red shift of its emission spectrum upon increase in membrane’s fluidity. GP has been used to measure this shift and therefore detect changes in lipids’ membrane phase state [35]. In fact, Laurdan GP has been found useful for numerous membrane studies [36, 37]. GP bears the same functional form as conventional fluorescence polarization:

$$GP=\frac{Ib-Ig}{Ib+Ig}$$ Eq 1

where I_b_ and I_g_ are the fluorescence intensities of Laurdan at blues and green filter, respectively.

### Calibration

The relative signal of the two filters (blue and green) was calibrated by using standard solutions, such as Laurdan in DMSO, with a GPref = 0.207. G-factor was then calculated according to published procedures and was used for correction of GP measurements [38].

### Imaging analysis

**Fig 1:**
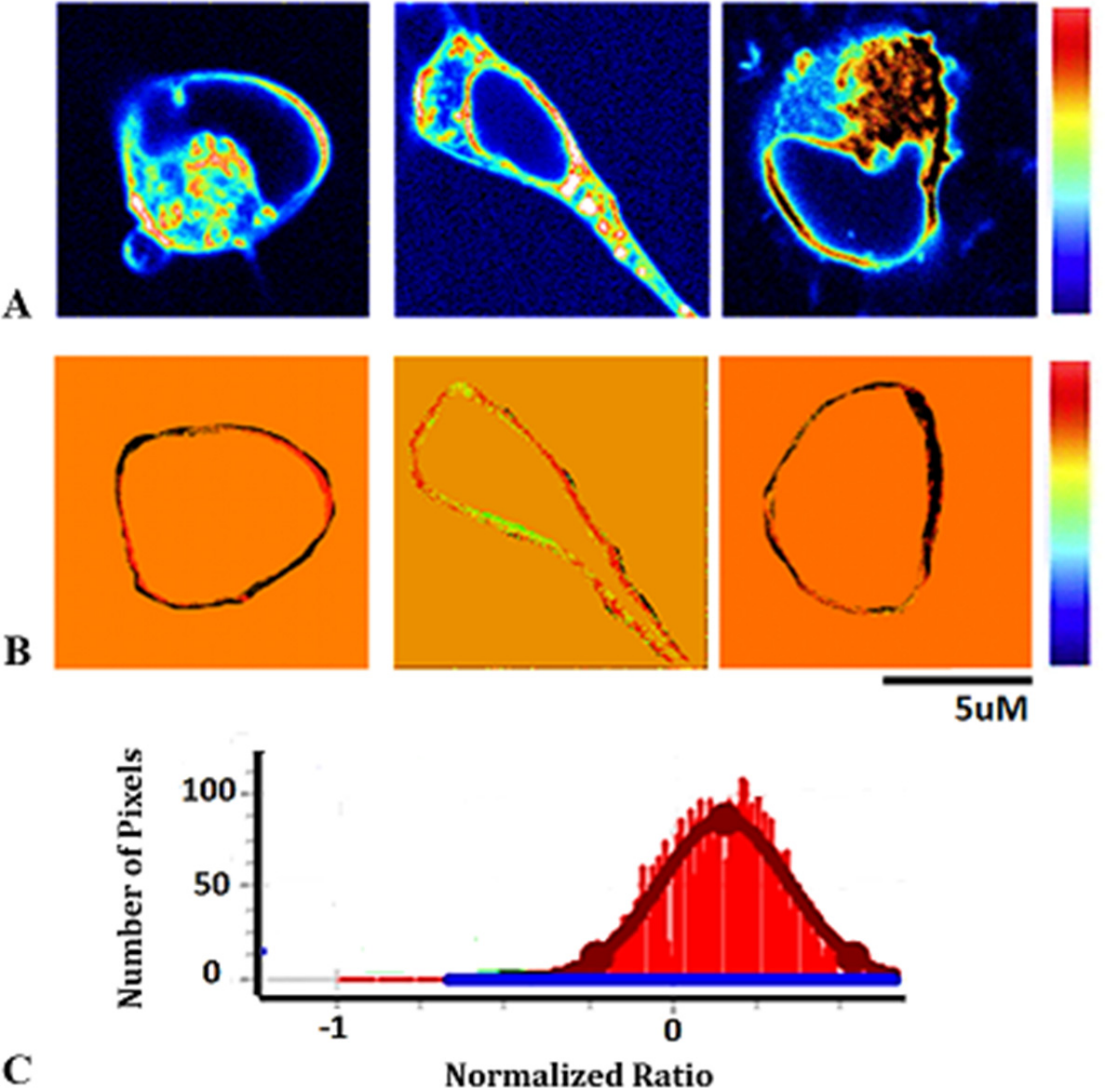
Laurdan GP analysis. A) Fluorescence-intensity images of three hN2 cells at 12 h observed in the blue channel (460–480). GP scale to pseudo color the intensity image is shown at the right. C) GP histogram from the corresponding image (membrane) in B). One Gaussian component is observed referring to the cell membrane after digital mask application. Average GP = 0.062.The width at half maximum is ~ 0.1.

Changes in membrane’s fluidity were determined using Generalized Polarization spectroscopic property of Laurdan. Data analysis was performed using the SimFCS software. Cell membranes were isolated from other cellular components by applying a digital mask as shown in (Fig 1B) and (S1B–S11B Figs). The Laurdan GP value reported here represents the average of the Gaussian fit of the histogram of GP values v/s pixels obtained from the Laurdan GP image. All values obtained correspond to the center of the Gaussian distribution. SimFCS has the option to display raw data as intensity images. We used RGB look-up table to show the images in a pseudo color format at which the color range is set to certain a value for a better contrast. Usually, pseudo colored pixels are merged with intensity images and saturated pixels can be detected by assigning a specific scale for displaying the image. Pixels with lowest intensity are colored blue and those with higher intensity are colored red. We set the saturation to 1 and the GP value as a color-coded image. We applied the “FRET ratio” routine of SimFCS (which is the calculation of the GP or normalized ratio) to exclude any saturated pixels from the analysis including the pixels arising from the growth media. Taking these parameters into consideration the GP image displayed will be devoid of low signal to noise pixels and contains GP as well as structural information. Hence the effect of media and background fluorescence will be excluded.

Three experiments were performed in each cell line at the indicated time points. Unpaired T-test was performed for statistical analysis which includes GP values from 10 different cells for each time point. P value <0.0001 is considered highly significant.

## Results

**Fig 2:**
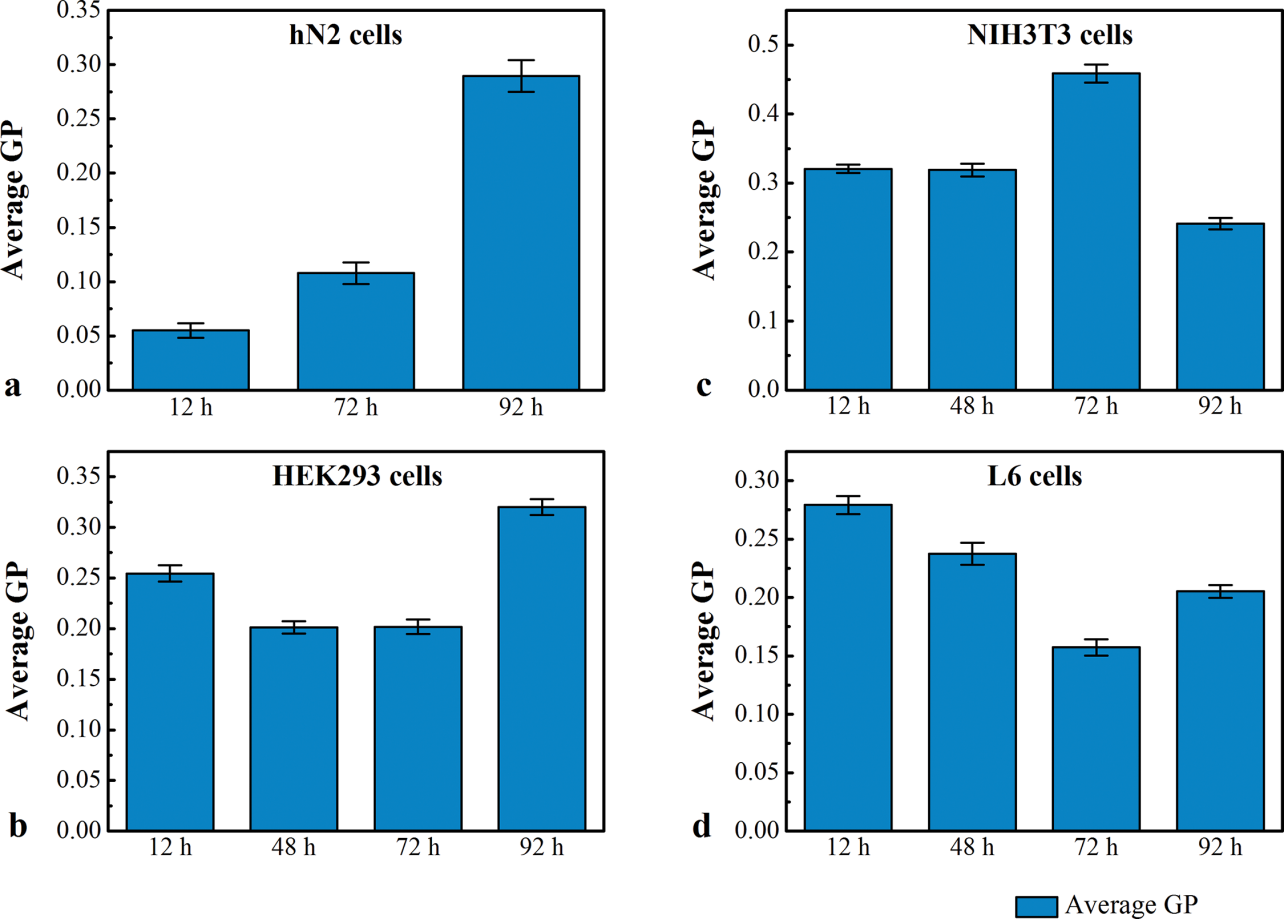
**GP distribution in a) hN2, b) HEK293, c) NIH3T3 and d) L6.** a) Histograms of GP distributions among hN2 cells at 12 h, 48 h, 72 h and 92 h respectively. At 72 h GP reached minimum values (0.17) ± SEM and increased to 0.28 ± SEM after 92 h. 10 cells were analyzed for each time point for statistical significance. b) Histograms of GP distributions among 10 HEK293 cells at 12 h, 48 h, 72 h and 92 h respectively. At 72 h GP values reached minimum values (0.2) ± SEM and increased to 0.327 ± SEM after 92 h. 10 cells were analyzed for each time point for statistical significance. c) Histograms of GP distributions among 10 NIH3T3 cells at 12 h, 48 h, 72 h and 92 h respectively. At 72 h GP reached maximum values (0.45) ± SEM and decreased to 0.25 ± SEM after 92 h. 10 cells were analyzed for each time point for statistical significance. d) Histograms of GP distributions among L6 cells at 12 h, 48 h, 72 h and 92 h respectively. At 72 h GP reached minimum values (0.17) ± SEM and increased to 0.22 ± SEM after 92 h. 10 cells were analyzed for each time point for statistical significance.

GP analysis in hN2 cells showed significant increase at 72 h and 92 h respectively compared to 12 h (Fig 2A) (hN2 12 h vs 72 h P = 4.50846E-08); (hN2 72 h vs 92 h P = 2.18126E-14); (hN2 12 h vs 92 h P = 2.2237E-17).

In HEK 293 cells the GP started to increase significantly only at 92 h compared to 72 h (Fig 2B) (HEK293 12 h vs 92 h P = 5.87941E-05); (HEK293 72 h vs 92 h P = 9.32247E-08).On the other hand, the period between 12 h and 72 h showed a very significant decrease in GP values (HEK293 12 h vs 72 h P = 0.000221). The increase in fluidity at the indicated time points may facilitate adhesion and fusion of HEK293 cells.

In NIH3T3 cells, membrane rigidity increased significantly to reach maximum values at 72 h (Fig 2C) (12 h vs 72 h P = 9.5689E-07) and drops at 92 h compared to 12 h and 72 h respectively (72 h vs 92 h P = 1.48909E-09); (12 h vs 92 h P = 9.18699E-06).

In L6 cells there was a significant decrease in GP values at 72 h compared to 12 h and a further increase at 92 h compared to 72 h (Fig 2D) (L6 12 h vs 72 h P = 6.93186E-12); (L6 12 h vs 92 h P = 1.69026E-08). The increase in membrane’s fluidity at 48 h and 72 h is probably correlated with the ability of L6 cells to adhere and fuse. It is well known that L6 cells grow in suspension and then start to adhere and fuse 48 h post-seeding.

**Fig 3:**
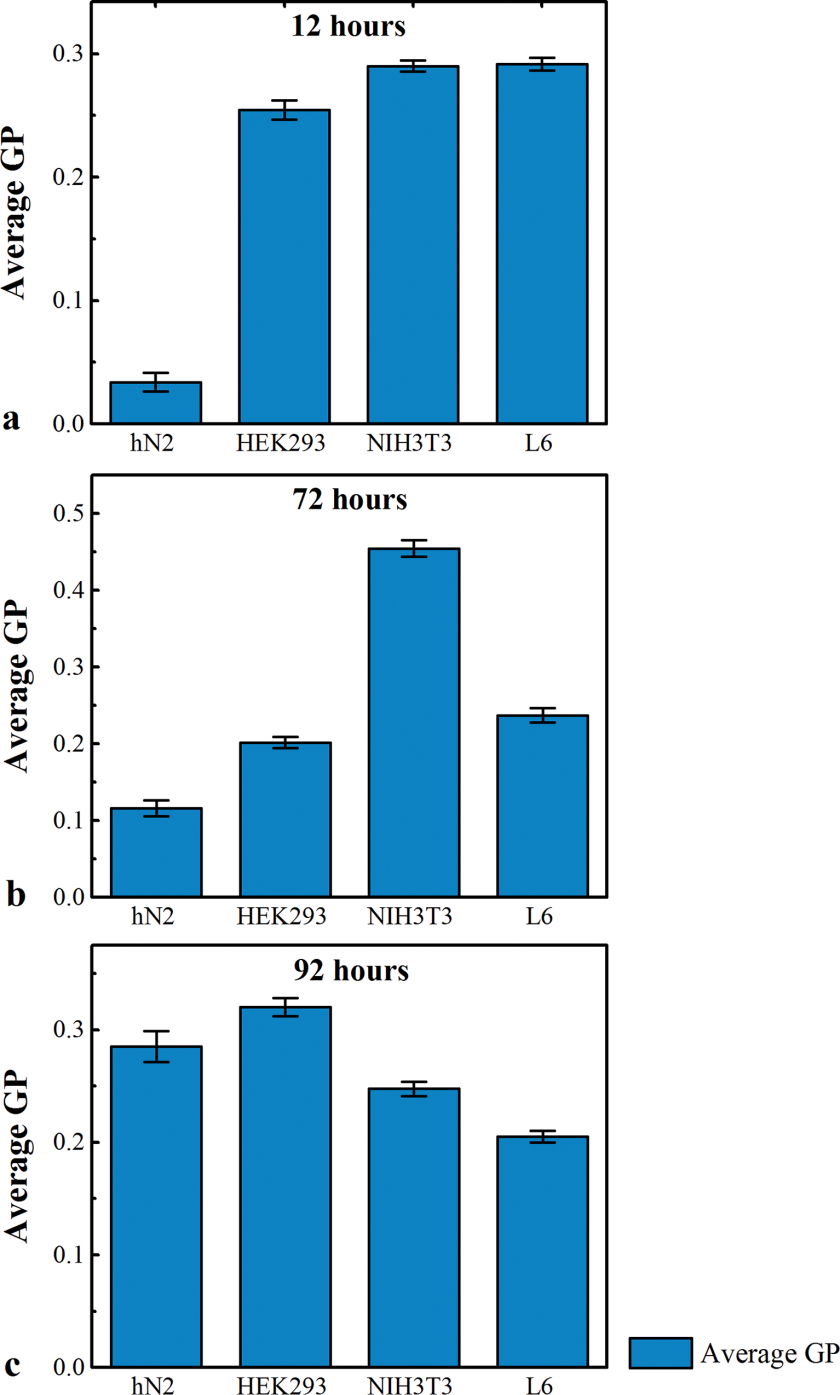
Summary of GP measurements. GP measurements in hN2, HEK293, NIH3T3 and L6 cells at a) 12 h; b) 72 h and c) 92 h.

When comparing GP measurements of the four cell lines hN2 cell are the ones with the lowest values indicating significant higher membrane fluidity at 12 h and 72 h respectively (Fig 3A and 3B). This might explain the need for these cells to undergo more potent morphological changes before maturing (12h hN2 vs 12 h HEK293 P = 4.205E-11); (12 h hN2 vs 12 h NIH3T3 P = 2.09233E-11) (P =; 12 h hN2 vs 12 h L6 P = 2.34631E-12); (72 h hN2 vs 72 h HEK293 P = 2.364E-07); (72 h hN2 vs 72 h NIH3T3 P = 4.23906E-10), (72 h hN2 vs 72 h L6 P = 0.0019537). At 12 h HEK293, NIH3T3 and L6 cells showed approximately close GP values (Fig 3A).

On the other hand, NIH3T3 cells have the highest GP values at 72h compared to the other cell lines (Fig 3B) (72 h NIH3T3 vs 72 h HEK293 P = 1.55102E-08); (72 h NIH3T3 vs 72 h L6 cells P = 2.4282E-08); (72 h NIH3T3 vs 72 h hN2 P = 4.23906E-10). This is expected because NIH3T3 have structural functions that necessitate a rigid membrane. At 92 h hN2, HEK293, NIH3T3 and L6 cells started to have similar GP values (Fig 3C). This period corresponds to a time where the cells have a stable structure.

In all monitored cell lines, the plasma membrane is not homogeneous. This is reflected on the width of the Gaussian distribution that represents the GP values of all pixels in a single cell membrane (Fig 1C and (S1C–S11C Figs in supplementary information). Spatially distributed domains of high and low GP values were observed. Therefore, in one cell the GP is not uniform. One possible explanation is the difference in the lipids’ composition. It is unlikely to have these fluctuations because of laser intensity variations or heating power of the laser beam. We are using very small excitation power that should not affect the GP measurements significantly due to heating effects of the scanning beam. This was described previously by Liu et al., 1994 [39].

**Statistical Analysis.** Unpaired T-test assuming unequal variance was performed for statistical analysis, which includes GP values from 10 different cells from three separate experiments for each time point. P value <0.0001 is considered extremely significant. Error bars represent standard error of the mean (SEM).

## Discussion

In this study we used 2 photon excitation microscopy to measure membrane fluidity in four cell lines (hN2, NIH3T3, HEK293 and L6) at different time points after seeding. Among all cell types investigated here, hN2 cells showed the highest membrane fluidity especially at earlier time points. They form adherent rounded clusters within 3h and then form networks with prominent neurites after 24 h until they make stable aggregates at 92 h. This drastic change in their morphology from circular to the neuron-like correlates with the increase in their GP values. Our results emphasize the role of membrane fluidity in structural plasticity, and its importance at the early times of development, probably due to the need of these cells for malleability during development. Cell membranes are composed of glycerophospholipids made up of several head groups linked via glycerol to two acyl chains which usually contain one unsaturated tail. Most eukaryotic cells have two more lipid classes: sterols (of which, cholesterol is a particular form) and sphingolipids [40]. Prokaryotes however are deprived of sterol which is known to play a key role in lipid packing and thus tune membrane rigidity and fluidity [41,42]. Both prokaryotes and eukaryotes share biophysical properties such as membrane fluidity. Despite this similarity, the plasma membrane of E-coli bacteria has a lower GP compared to human RBC membrane probably due to the lack of cholesterol in the former. Furthermore, isolates of bacterial membrane lipids have negative GP values, indicating the involvement of proteins in rigidifying the cell membrane whereas lipids are the major contributors in decreasing membrane rigidity among eukaryotes [43]. Sterols are present at high levels in plasma membrane and at low levels in internal membranes such as endoplasmic reticulum (ER) and Golgi [44]. Using phasor analysis Bonaventura et al. (2013) have shown the existence of regions of different membrane’s fluidity in E6 and E12 neuronal precursor cells. This is probably due to the difference in their lipid composition which fluctuates laterally provoking the average membrane fluidity to be similar at the two indicated time points [45]. Researchers showed that cholesterol modifies phospholipid bilayer phase domain properties, its water concentration and dynamics [46,47]. Its implication in the pathogenesis of neurodegenerative diseases was also assessed. For instance, young neurons (7 days old) have lower membrane cholesterol levels than those detected in 21-days old neurons followed by amyloid betta (Aβ) treatment in an *in vitro* Alzheimer’s disease model [48]. Also mature hippocampal neurons showed higher membrane cholesterol levels compared to juvenile ones as shown by filipin fluorescence measurement [49]. Although our data on membrane fluidity focused on neurons that are just few days old, this could be an indication that neurons acquire quite rigid membranes upon maturity. Our results on hN2 cells might shed a light on the effect of membrane fluidity on cellular adhesion and differentiation. This high rigidity facilitates the anchoring of cytoskeletal proteins to the membrane in a regulated manner. Membrane stiffness was also shown to be effective in other cell types and during several pathological conditions. For instance, in type-2 diabetes concomitant increase of erythrocyte membrane stiffness may decrease the microcirculatory flow and lead to tissue hypoxia and insufficient tissue nutrition. It has been speculated that lipid molecules may influence glucose transport by the insulin-independent GLUTs and in turn glucose effectiveness [50]. Also membrane’s fluidity changes in certain types of cancer allowing the cells to adapt to their new demands such as an increase in leakage. It has been found that membrane fluidity can be used as a prognostic tool in lung cancer [51] as it helps in lung colonization potential, metastasis and tumor cell motility [52,53]

In L6 (myoblasts), cells membrane fluidity increases with time but their membrane is more rigid at 12 h compared to later time points (48 h and 92 h). These cells grow partially in suspension until they form an adherent layer within 48 h. The higher membrane rigidity at 12 h can be attributed to their growth pattern as they are still in a transition state between a semi-adherent and an adherent form. L6 cells fuse in culture to form multinucleated myotubes and striated fibers 48h after seeding. It is known that the extent of cell fusion decreases with time (after 72 h) and with passage number. Interestingly this was not attributed to any fusion-associated induction of surface proteins although microsomal fraction analysis showed a fusion-associated 38-kDa glycopolypeptide [54]. Furthermore, various novel cytoskeletal events are involved in myoblast fusion such as actin and non-muscle myosin IIA (NM-MHC-IIA) [55]. During the fusion of myoblasts it was found that few lipids such as inositol are broken down while others, such as diacylglycerol and phosphatidic acid, are synthesized [56,57].

The study of membrane’s fluidity in L6 cells helps better understand their membrane fusion mechanism. At early time points (12 h) when cells have not yet fused, their cell membrane is still rigid and probably preventing cytoskeletal proteins from interdigitating through it or into the lipid rafts in particular. As their ability to adhere and fuse increases with time (48 h onwards) their membrane fluidity also increases drastically to reach maximal values at 72 h. However, at 92 h membranes become even more rigid, a time when their cell fusion tendency also decreases as shown from previous studies [58] supporting our findings that indicate a strong correlation between their time dependent morphological changes and their membrane’s fluidity. In conclusion, the differentiation of skeletal muscle cells requires myoblast fusion in a tightly programmed manner. Although lipids and proteins modification is speculated in the process, their function and contribution remains unclear. Our results emphasize that fluidity and cellular fusion are required during cell development. Our preliminary results beg to understand the mechanism of recovery during muscular injury where satellite cells fuse in the same manner as the embryonic development of muscle and the effect of membrane’s rigidity in this recovery mechanism such as the effect of a rigidifying molecule like cholesterol.

NIH3T3 cells (fibroblasts) are the most common type of cells in the animal connective tissue that synthesize the extracellular matrix and glycogen which forms the structural framework (stroma) for animal tissues. Compared to other cell types studied here, they have the highest membrane rigidity at 72h. Their GP values increase with time indicating a probable correlation between cellular plasticity and membrane fluidity. In HEK293 cells, membrane fluidity increased with time to reach maximal values at 72 h. After 92 h the cell membrane becomes more rigid probably indicating a stabilization process because the cells are already fused and developed. Furthermore, these cells take few hours before adhering to the flask and it takes them around 24 h before changing from round to polygonal shapes. We noted however that at 12 h the cells are still not strongly attached to the flask which might be a reason of their high membrane rigidity.

## Conclusion

The variations in GP values among different cell lines studied above can be an indication of the difference in their lipid composition. It is known that the lipid composition of different subcellular fractions varies within and among different organs. For instance, the brain has the highest percentage of phosphatidyl serine and lowest percentage of diphosphatadyl glycerol. Its major lipid content comes from the plasma membrane. Our GP and stiffness measurements on neurons, unlike other cells studied here, showed that they exhibited a substantial change in their membrane stiffness. We can hypothesize that neurons and glia needs to form surface extensions and long neurites and thus might require a larger amount of lipids bulk to be available at their surface conveyed from the mitochondria [59]. Also, during brain development there are marked changes in glycoconjugates expression ranging from complex proteoglycans to gangliosides [60,61]. In old humans and in some diseases (>70 years) the total amount of brain ganglioside decreases significantly [62]. For example, brain insulin resistance induced by aging and peripheral insulin resistance is accompanied by enhanced assembly of Aβ fibrils on neuronal surface due to increased clustering of GM1 in their membrane lipid rafts which might affect the stiffness of their cell membrane [48]. We showed that membrane’s fluidity depends on the cell type and age and correlates with the aforementioned studies. It sheds a light on the importance of membrane fluidity during cellular development. GP measurements obtained via a 2-photon confocal microscopy gives a clear picture on the trend of fluidity changes. Lipid membranes have been extensively studied because they are model of all biological materials and are accessible models for random surfaces [63,64,65,66], but there is still a need for a thorough analysis in live cells or tissues apart from invasive techniques which rely on membrane isolation which may lead to artifacts. We are the first to use a systematic analysis of the GP values to study the mechanical state of the cell membranes of four cell lines. Although our results give an average measurements of the state of the membranes, their lipid heterogeneity should be addressed. Modulation of the cholesterol in lipid rafts and consequently their fluidity opens the opportunity for the cure of certain diseases. A better understanding of how lipid rafts regulate cell destination, and how membrane domains can be adjusted during cellular development, would lead to an improvement in inventing novel strategies for disease treatment. Therefore extending the current study to cover various brain regions at different gestational ages is one of our future aims. Determining the causative relationship between fatty acid composition of cell membranes and certain disease stage such as cancer or neurodegeneration will contribute to the basic understanding of pathophysiological mechanisms of these diseases.

## Supporting Information

S1 Fig Laurdan GP analysis in 72 h old hN2 cells. A) Fluorescence-intensity images of three hN2 cells at 72 h observed in the blue channel (460–480). GP scale to pseudo color the intensity image is shown at the right. C) GP histogram from the corresponding image in B). One Gaussian component is observed referring to the cell membrane after digital mask application. Average GP = 0.147. The width at half maximum is ~ 0.25.
(TIF)

S2 Fig Laurdan GP analysis in 92 h old hN2 cells. A) Fluorescence-intensity images of three hN2 cells at 92 h observed in the blue channel (460–480). GP scale to pseudo color the intensity image is shown at the right. C) GP histogram from the corresponding image in B). One Gaussian component is observed referring to the cell membrane after digital mask application. Average GP = 0.338. The width at half maximum is ~ 0.45.
(TIF)

S3 Fig Laurdan GP analysis in 12 h old NIH3T3 cells. A) Fluorescence-intensity images of three NIH3T3 cells at 12 h observed in the blue channel (460–480). GP scale to pseudo color the intensity image is shown at the right. C) GP histogram from the corresponding image in B). One Gaussian component is observed referring to the cell membrane after digital mask application. Average GP = 0.280. The width at half maximum is ~ 0.45.
(TIF)

S4 Fig Laurdan GP analysis in 72 h old NIH3T3 cells. A) Fluorescence-intensity images of three NIH3T3 cells at 72 h observed in the blue channel (460–480). GP scale to pseudo color the intensity image is shown at the right. C) GP histogram from the corresponding image in B). One Gaussian component is observed referring to the cell membrane after digital mask application. Average GP = 0.485. The width at half maximum is ~ 0.75.
(TIF)

S5 Fig Laurdan GP analysis in 92 h old NIH3T3 cells. A) Fluorescence-intensity images of three NIH3T3 cells at 92 h observed in the blue channel (460–480). GP scale to pseudo color the intensity image is shown at the right. C) GP histogram from the corresponding image in B). One Gaussian component is observed referring to the cell membrane after digital mask application. Average GP = 0.263. The width at half maximum is ~ 0.39.
(TIF)

S6 Fig Laurdan GP analysis in 12h old HEK293 cells. A) Fluorescence-intensity images of three HEK293 cells at 12 h observed in the blue channel (460–480). GP scale to pseudo color the intensity image is shown at the right. C) GP histogram from the corresponding image in B). One Gaussian component is observed referring to the cell membrane after digital mask application. Average GP = 0.242. The width at half maximum is ~ 0.5.
(TIF)

S7 Fig Laurdan GP analysis in 72 h old HEK293 cells. A) Fluorescence-intensity images of three HEK293 cells at 72 h observed in the blue channel (460–480). GP scale to pseudo color the intensity image is shown at the right. C) GP histogram from the corresponding image in B). One Gaussian component is observed referring to the cell membrane after digital mask application. Average GP = 0.194. The width at half maximum is ~ 0.35.
(TIF)

S8 Fig Laurdan GP analysis in 92 h old HEK293 cells. A) Fluorescence-intensity images of three HEK293 cells at 92 h observed in the blue channel (460–480). GP scale to pseudo color the intensity image is shown at the right. C) GP histogram from the corresponding image in B). One Gaussian component is observed referring to the cell membrane after digital mask application. Average GP = 0.366. The width at half maximum is ~ 0.566.
(TIF)

S9 Fig Laurdan GP analysis in 12 h old L6 cells. A) Fluorescence-intensity images of three L6 cells at 12 h observed in the blue channel (460–480). GP scale to pseudo color the intensity image is shown at the right. C) GP histogram from the corresponding image in B). One Gaussian component is observed referring to the cell membrane after digital mask application. Average GP = 0.307. The width at half maximum is ~ 0.45.
(TIF)

S10 Fig Laurdan GP analysis in 72 h old L6 cells. A) Fluorescence-intensity images of three L6 cells at 72 h observed in the blue channel (460–480). GP scale to pseudo color the intensity image is shown at the right. C) GP histogram from the corresponding image in B). One Gaussian component is observed referring to the cell membrane after digital mask application. Average GP = 0.152. The width at half maximum is ~ 0.25.
(TIF)

S11 Fig Laurdan GP analysis in 92 h old L6 cells. A) Fluorescence-intensity images of three L6 cells at 92 h observed in the blue channel (460–480).GP scale to pseudo color the intensity image is shown at the right. C) GP histogram from the corresponding image in B). One Gaussian component is observed referring to the cell membrane after digital mask application. Average GP = 0.225. The width at half maximum is ~ 0.375.
(TIF)
