# Supplementary figures and images for: Assessment of Membrane Fluidity Fluctuations during Cellular Development Reveals Time and Cell Type Specificity
Source: PLoS One. 2016 Jun 30;11(6):e0158313. doi: 10.1371/journal.pone.0158313 (PMC4928918; doi:10.1371/journal.pone.0158313)

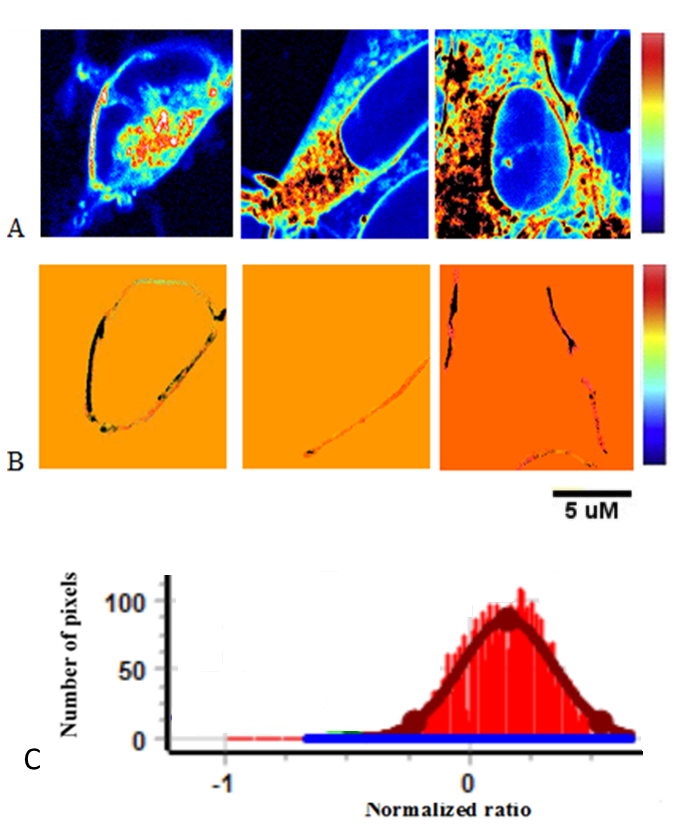

Supplement: S1 Fig — A) Fluorescence-intensity images of three hN2 cells at 72 h observed in the blue channel (460–480). GP scale to pseudo color the intensity image is shown at the right. C) GP histogram from the corresponding image in B). One Gaussian component is observed referring to the cell membrane after digital mask application. Average GP = 0.147. The width at half maximum is ~ 0.25. (TIF) [file pone.0158313.s001.tif]

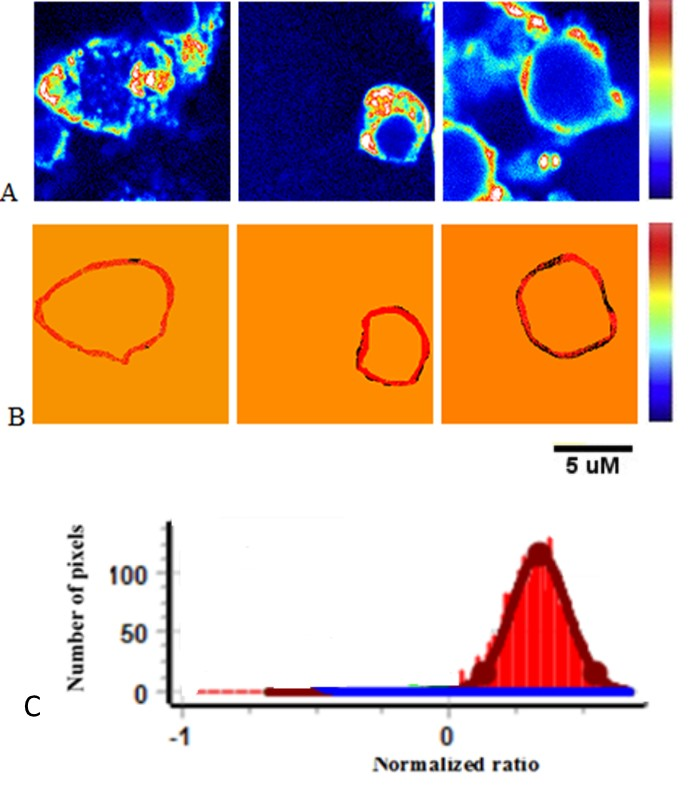

Supplement: S2 Fig — A) Fluorescence-intensity images of three hN2 cells at 92 h observed in the blue channel (460–480). GP scale to pseudo color the intensity image is shown at the right. C) GP histogram from the corresponding image in B). One Gaussian component is observed referring to the cell membrane after digital mask application. Average GP = 0.338. The width at half maximum is ~ 0.45. (TIF) [file pone.0158313.s002.tif]

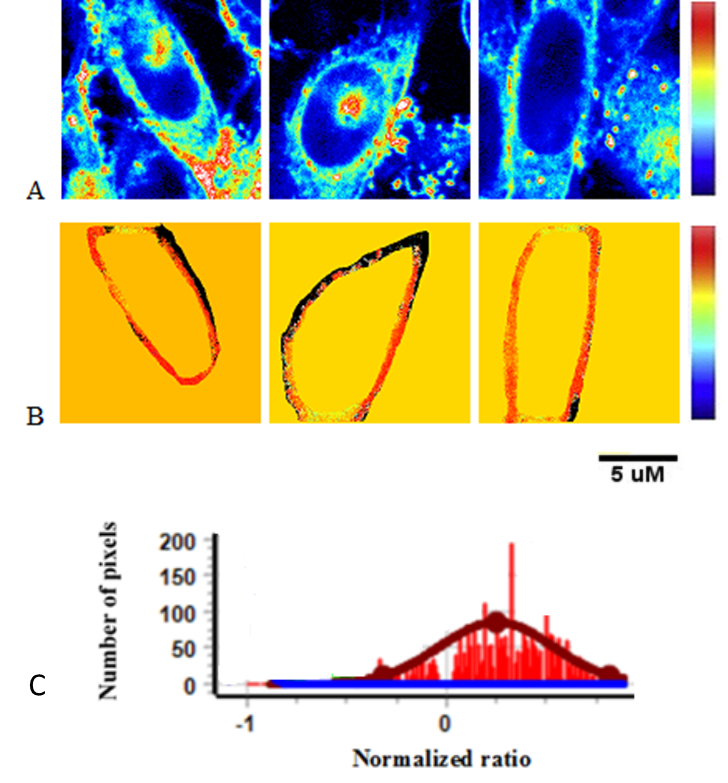

Supplement: S3 Fig — A) Fluorescence-intensity images of three NIH3T3 cells at 12 h observed in the blue channel (460–480). GP scale to pseudo color the intensity image is shown at the right. C) GP histogram from the corresponding image in B). One Gaussian component is observed referring to the cell membrane after digital mask application. Average GP = 0.280. The width at half maximum is ~ 0.45. (TIF) [file pone.0158313.s003.tif]

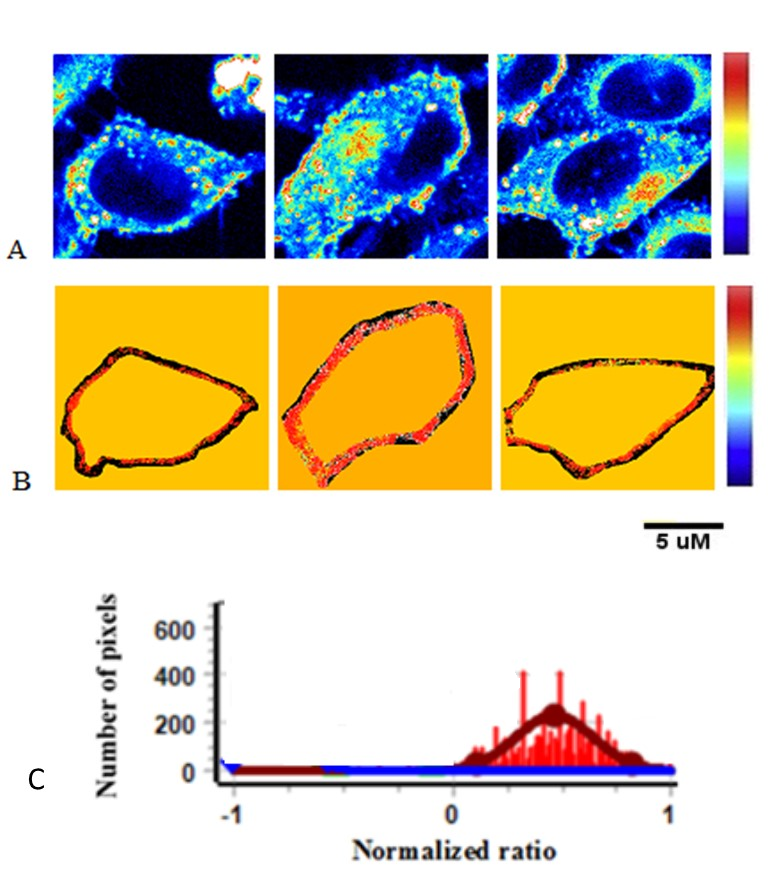

Supplement: S4 Fig — A) Fluorescence-intensity images of three NIH3T3 cells at 72 h observed in the blue channel (460–480). GP scale to pseudo color the intensity image is shown at the right. C) GP histogram from the corresponding image in B). One Gaussian component is observed referring to the cell membrane after digital mask application. Average GP = 0.485. The width at half maximum is ~ 0.75. (TIF) [file pone.0158313.s004.tif]

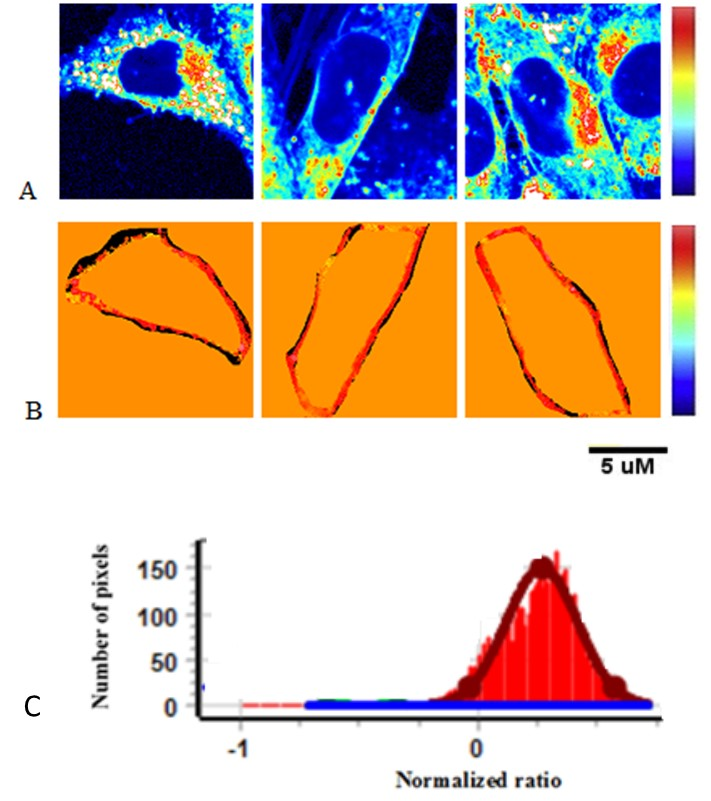

Supplement: S5 Fig — A) Fluorescence-intensity images of three NIH3T3 cells at 92 h observed in the blue channel (460–480). GP scale to pseudo color the intensity image is shown at the right. C) GP histogram from the corresponding image in B). One Gaussian component is observed referring to the cell membrane after digital mask application. Average GP = 0.263. The width at half maximum is ~ 0.39. (TIF) [file pone.0158313.s005.tif]

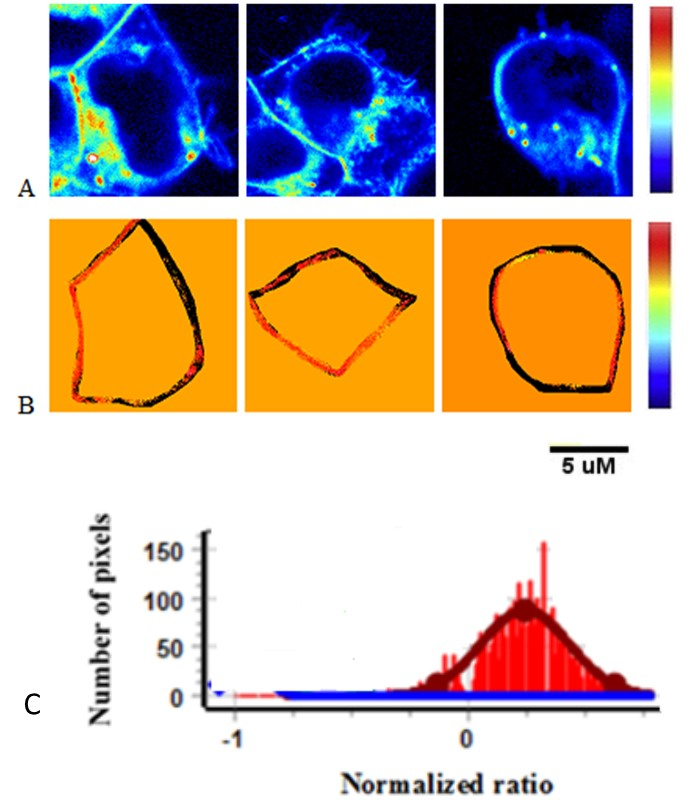

Supplement: S6 Fig — A) Fluorescence-intensity images of three HEK293 cells at 12 h observed in the blue channel (460–480). GP scale to pseudo color the intensity image is shown at the right. C) GP histogram from the corresponding image in B). One Gaussian component is observed referring to the cell membrane after digital mask application. Average GP = 0.242. The width at half maximum is ~ 0.5. (TIF) [file pone.0158313.s006.tif]

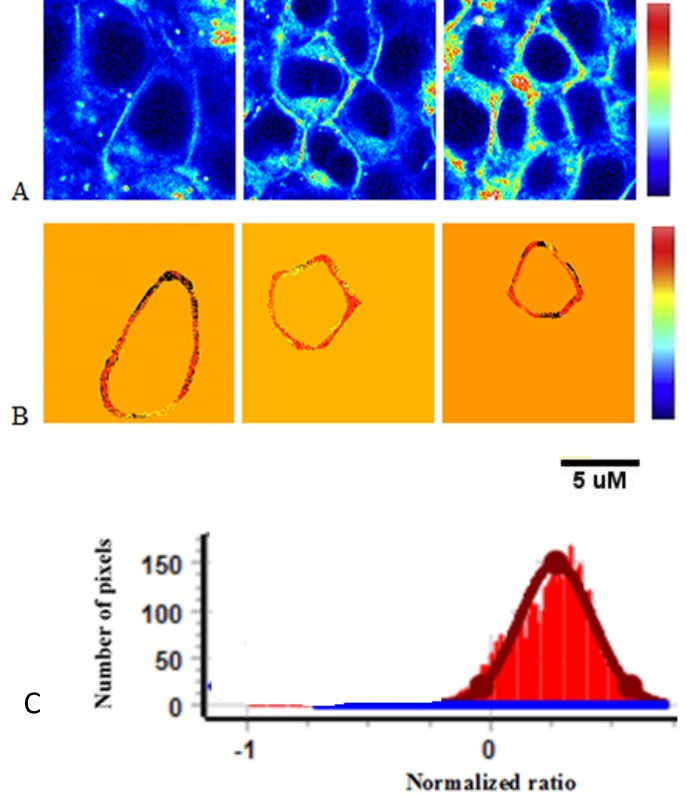

Supplement: S7 Fig — A) Fluorescence-intensity images of three HEK293 cells at 72 h observed in the blue channel (460–480). GP scale to pseudo color the intensity image is shown at the right. C) GP histogram from the corresponding image in B). One Gaussian component is observed referring to the cell membrane after digital mask application. Average GP = 0.194. The width at half maximum is ~ 0.35. (TIF) [file pone.0158313.s007.tif]

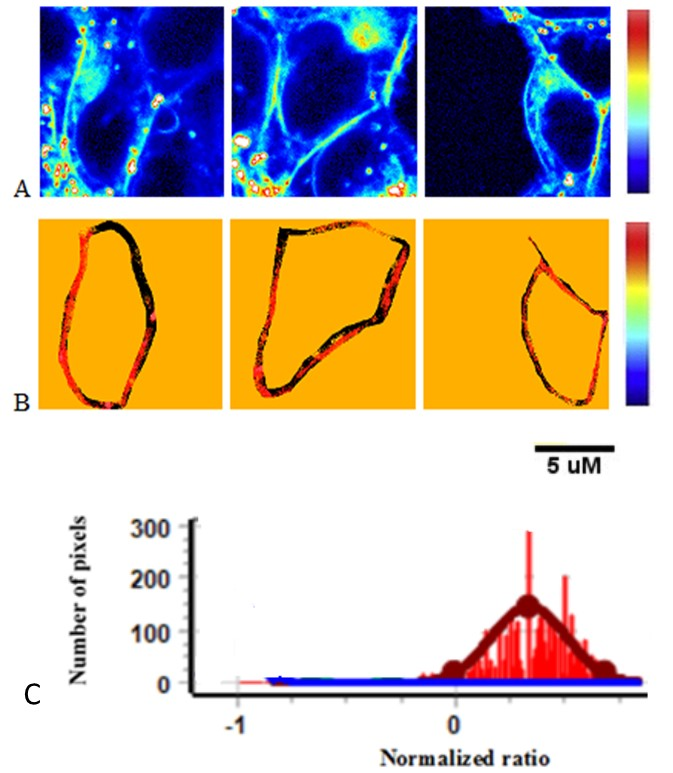

Supplement: S8 Fig — A) Fluorescence-intensity images of three HEK293 cells at 92 h observed in the blue channel (460–480). GP scale to pseudo color the intensity image is shown at the right. C) GP histogram from the corresponding image in B). One Gaussian component is observed referring to the cell membrane after digital mask application. Average GP = 0.366. The width at half maximum is ~ 0.566. (TIF) [file pone.0158313.s008.tif]

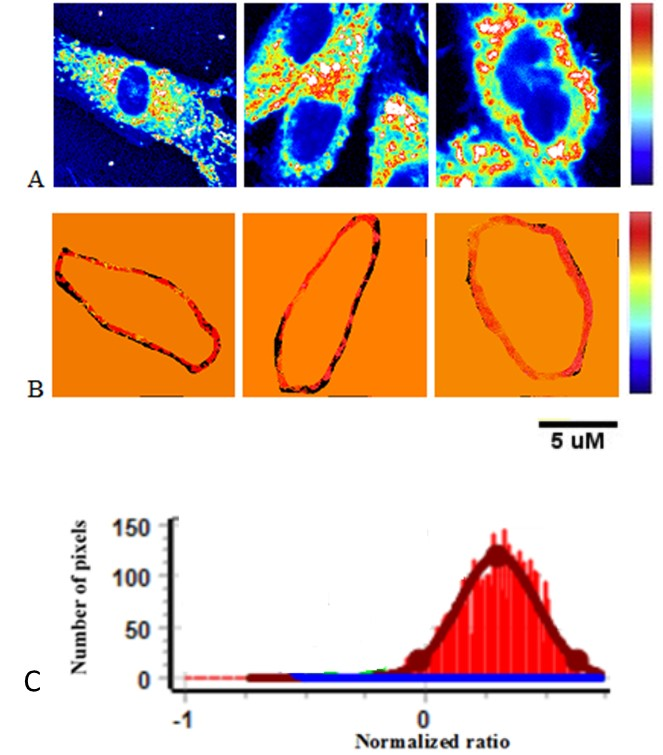

Supplement: S9 Fig — A) Fluorescence-intensity images of three L6 cells at 12 h observed in the blue channel (460–480). GP scale to pseudo color the intensity image is shown at the right. C) GP histogram from the corresponding image in B). One Gaussian component is observed referring to the cell membrane after digital mask application. Average GP = 0.307. The width at half maximum is ~ 0.45. (TIF) [file pone.0158313.s009.tif]

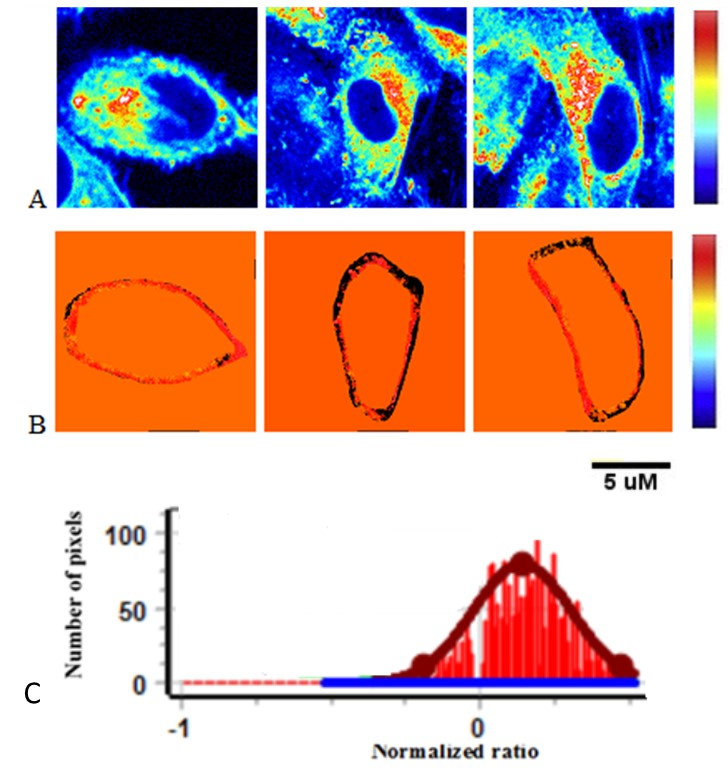

Supplement: S10 Fig — A) Fluorescence-intensity images of three L6 cells at 72 h observed in the blue channel (460–480). GP scale to pseudo color the intensity image is shown at the right. C) GP histogram from the corresponding image in B). One Gaussian component is observed referring to the cell membrane after digital mask application. Average GP = 0.152. The width at half maximum is ~ 0.25. (TIF) [file pone.0158313.s010.tif]

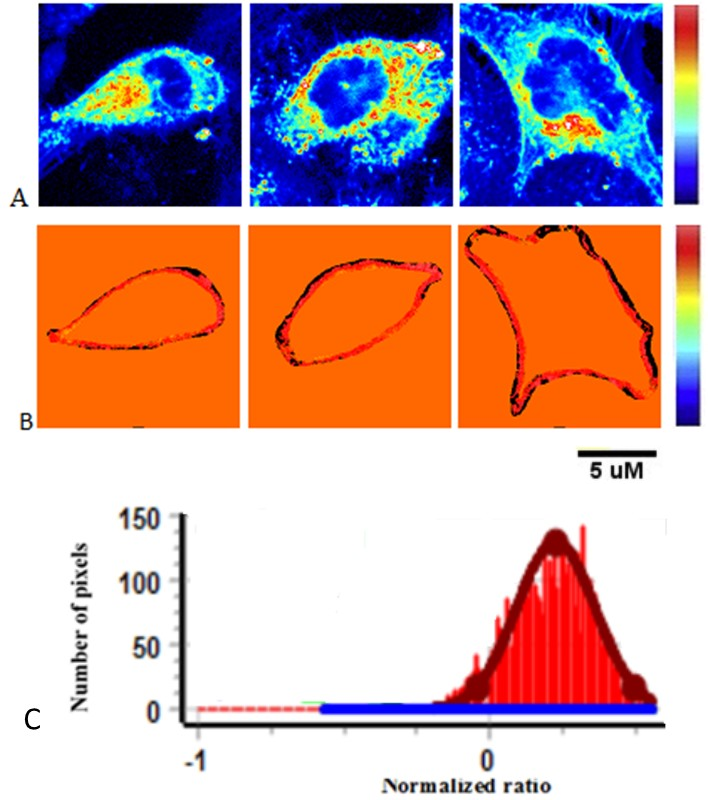

Supplement: S11 Fig — A) Fluorescence-intensity images of three L6 cells at 92 h observed in the blue channel (460–480).GP scale to pseudo color the intensity image is shown at the right. C) GP histogram from the corresponding image in B). One Gaussian component is observed referring to the cell membrane after digital mask application. Average GP = 0.225. The width at half maximum is ~ 0.375. (TIF) [file pone.0158313.s011.tif]
